# Estimating Spatio-Temporal Dynamics of *Aedes Albopictus* Dispersal to Guide Control Interventions in Case of Exotic Arboviruses in Temperate Regions

**DOI:** 10.1038/s41598-019-46466-4

**Published:** 2019-07-16

**Authors:** Francesca Marini, Beniamino Caputo, Marco Pombi, Manuela Travaglio, Fabrizio Montarsi, Andrea Drago, Roberto Rosà, Mattia Manica, Alessandra della Torre

**Affiliations:** 1 Dipartimento di Sanità Pubblica e Malattie Infettive, “Sapienza” Università di Roma, Piazzale Aldo Moro 5, 00185 Rome, Italy; 2 Dipartimento di Biologia, Università di Padova, Viale G. Colombo 3, 35121 Padua, Italy; 3 Istituto Zooprofilattico Sperimentale delle Venezie, Viale dell’Università 10, 35020 Legnaro, (PD) Italy; 4 ENTOSTUDIO srl, Viale del Lavoro 66, 35020 Ponte San Nicolò, (PD) Italy; 5 Department of Biodiversity and Molecular Ecology, Research and Innovation Centre, Fondazione Edmund Mach, Via E. Mach 1, 38010 San Michele all’Adige, Italy; 6 Center Agriculture Food Environment, University of Trento, 38010 San Michele all’Adige, Trento Italy; 7 Present Address: Biotechnology and Biological Control Agency (BBCA) Onlus, Via Angelo Signorelli 105, 00123 Rome, Italy

**Keywords:** Ecological modelling, Invasive species

## Abstract

The increasing number of exotic arbovirus cases imported in Europe and the 2017 chikungunya outbreak in central/southern Italy highlight the urgency of evidence-based outbreak management plans to predict, prevent or interrupt spreading of these arboviruses to non-endemic countries in temperate regions. We here present the results of three mark-release-recapture experiments conducted in a peri-urban area of North-East Italy to estimate the spatio-temporal dynamics of the dispersal of *Aedes albopictus* females looking for oviposition sites. The Flight Range of 90% of the mosquito population (FR_90_) was found to exceed 200 m, consistently with data obtained from a previous study conducted in a highly urbanised area in Rome (Central Italy). Modelling results showed that dispersal can be so rapid that insecticide spraying within a 200m-radius around a potential infected case leaves >10% probability that a potentially infected mosquito escapes the treatment, even if this is carried out after only 2–3 days since the importation of a viremic case. These data provide evidence in favour of an update of guidelines for the control of exotic autochthonous arbovirus transmission in temperate areas and highlight the need of effective surveillance approaches and rapid response to contain the risks associated to imported viremic cases.

## Introduction

Knowledge about the spatio-temporal dynamics of dispersal of adult mosquito vectors of diseases is instrumental for understanding mosquito-borne disease transmission dynamics and for effectively calibrating control intervention in order to prevent or interrupt pathogen transmission. Active dispersal of adult mosquitoes is triggered by the need to find mates, sugar sources, resting sites and, in the case of females, hosts for blood-meals and oviposition sites and is highly affected by intrinsic species-specific flight capability, as well as by ecological (e.g. abundance and location of sugar sources, hosts, resting sites and oviposition sites) and climatic (e.g. temperature, rainfall, light intensity, wind speed and direction at ground level) conditions^1^. Reliable estimates of active mosquito dispersal and flight ranges are thus very hard to obtain. This is even more challenging in the case of exophagic and exophilic species which are particularly difficult to recapture in the frame of Mark–Release–Recapture (MRR) studies^1^.

For the above reasons, little is known about the flight range of *Aedes albopictus*, the Asian Tiger Mosquito, the species which in the last 30 years has invaded all continents (except Antarctica) thanks to the passive transportation of its eggs mostly inside used tires and lucky bamboos, and has stably colonized not only tropical, but also temperate regions due to the capacity to overcome the cold months in a state of embryonic hibernation^2^. These capacities have turned the species into one of the most successful invasive animal species worldwide^3^ and in a global public health threat due to its competence to transmit a large number of exotic arboviruses (such as Chikungunya (CHIKV), Dengue (DENV),^4^ and Zika^5^). This is already testified by *Ae. albopictus* central role in large CHIKV epidemics in Indian Ocean in 2006–07^6^ and in the Caribbean and Americas since 2015^7^ and in several cases of autochthonous transmission of CHIKV and DENV in temperate countries^8^.

Few MRR studies have been carried out to investigate the capacity to disperse of both host-seeking gravid *Ae. albopictus* females looking for a host and blood-fed females looking for oviposition sites. In the former case, most marked host-seeking females were collected within 100 m from the release site (Hawaii^9^, Missouri^10^, La Réunion Island^11^), while investigation carried out in Texas, on host-seeking females emerged from larvae marked with stable isotopes, showed that approximately 79% of them were found within 250 m from their natal site and all of them remained within a 1 km distance^12^. However, females released in the forest were shown to flight up to 1 km to reach an urban area with high densities of possible hosts^13^. In the case of oviposition-associated dispersal, studies exploiting the release of Rb-labelled females and recapture of Rb-marked eggs reported presence of Rb-marked eggs in ovitraps throughout the whole 160 m radius urban area sampled in Singapore^14^, while in Brazil most Rb-marked eggs and fluorescent dusted females were found within 100–200 m from a release site^13,15^. In Florida, Davis *et al*.^16^ showed that self-marked gravid *Ae. albopictus* female dispersal increased with time, but seemed to stabilize around 90 m from the initial marking site.

In 2008, we carried out 3 MRR experiments within a highly urbanised area in the city of Rome (central Italy) to analyse dispersal of florescent dusted *Ae. albopictus* females released after blood-meal and recaptured by sticky traps (STs), and obtained flight ranges of 90% of the released females ranging between 168 and 236 m^17^. The first aim of the present study - carried out with the same design, but in a peri-urban area in north-east Italy - was to understand whether the flight ranges assessed in Rome could represent a common feature of *Ae. albopictus* female dispersal associated to search of an oviposition site in temperate regions. In addition, we exploited the data to model the temporal dynamics of the dispersal. This parameter has been largely neglected in previous studies, despite its high relevance for models estimating the dynamics of spread of autochthonous transmission of exotic arboviruses in temperate regions, as well as for the calibration of the spatial/temporal scale of insecticide treatments to be implemented to prevent or interrupt spread of arbovirus outbreaks. In fact, the recent cases of autochthonous CHIKV and DENV transmission in Europe^8^ highlight the urgency and timeliness to improve preparedness to predict, prevent or interrupt spreading of these and other exotic tropical arboviruses to non-endemic northern countries.

## Results

### Mark-release-recapture experiments

A total of 356 marked *Ae. albopictus* females were collected out of the 3,959 released ones (MRR1 = 101/1,149, MRR2 = 214/1,600; MRR3 = 41/1,210; Supplementary Table S2). In addition, 16,938 wilds *Ae. albopictus* were collected: 79.3% females (MRR1 = 3,607; MRR2 = 7,065; MRR3 = 2,754), 14.4% males (MRR1 = 669; MRR2 = 1,533; MRR3 = 234), while for the remaining 6.4% (MRR1 = 250; MRR2 = 401; MRR3 = 425) was not possible to determine the gender, due to bad preservation of carcasses glued on the STs. Some specimens, mostly females, of *Culex* sp.*, Culiseta* sp.. and *Aedes caspius* were also found in the STs but not counted.

The recapture rates at 11 days from release were 8.8% (MRR1), 12.9% (MRR2), 3.0% (MRR3) and significantly different among the three experiments (Supplementary Table S1; comparison between GLMs with or without releases as covariate: GLM deviance = 96.5, df = 2, P < 0.0001). Recapture rates observed in MRR1 and MRR2 were higher than those observed in the study carried out with a similar experimental design in a smaller study area (i.e. 250 m radius *vs* 500 m radius, 55 STs *vs* 96 STs) in Rome (*i.e*. the odds of recapturing a marked mosquito was 0.45 lower in Rome with a confidence interval of 0.39–0.59; Binomial GLM: deviance 39.1, p-value < 0.001). This may be due to the larger sampling area and the higher number of STs used in the present study as opposed to Rome and/or to an inherent bias in using STs as recapture method (i.e. the power of recapture is dependent on the competition with other natural oviposition sites in the study area, usually quite abundant in urban areas). However, it is worth to note that the recapture rates obtained in both studies are generally higher than those obtained in MRR studies on *Ae. albopictus* females dispersal after blood-meal carried out with different recapture approaches^9,10,13^. These results confirm that STs are a valuable tool for MRR studies on *Ae. albopictus* ovipositing or resting females (see also Marini *et al*.^17^ for a detailed discussion on pros and cons of using STs in the context of MRR studies).

**Figure 1:**
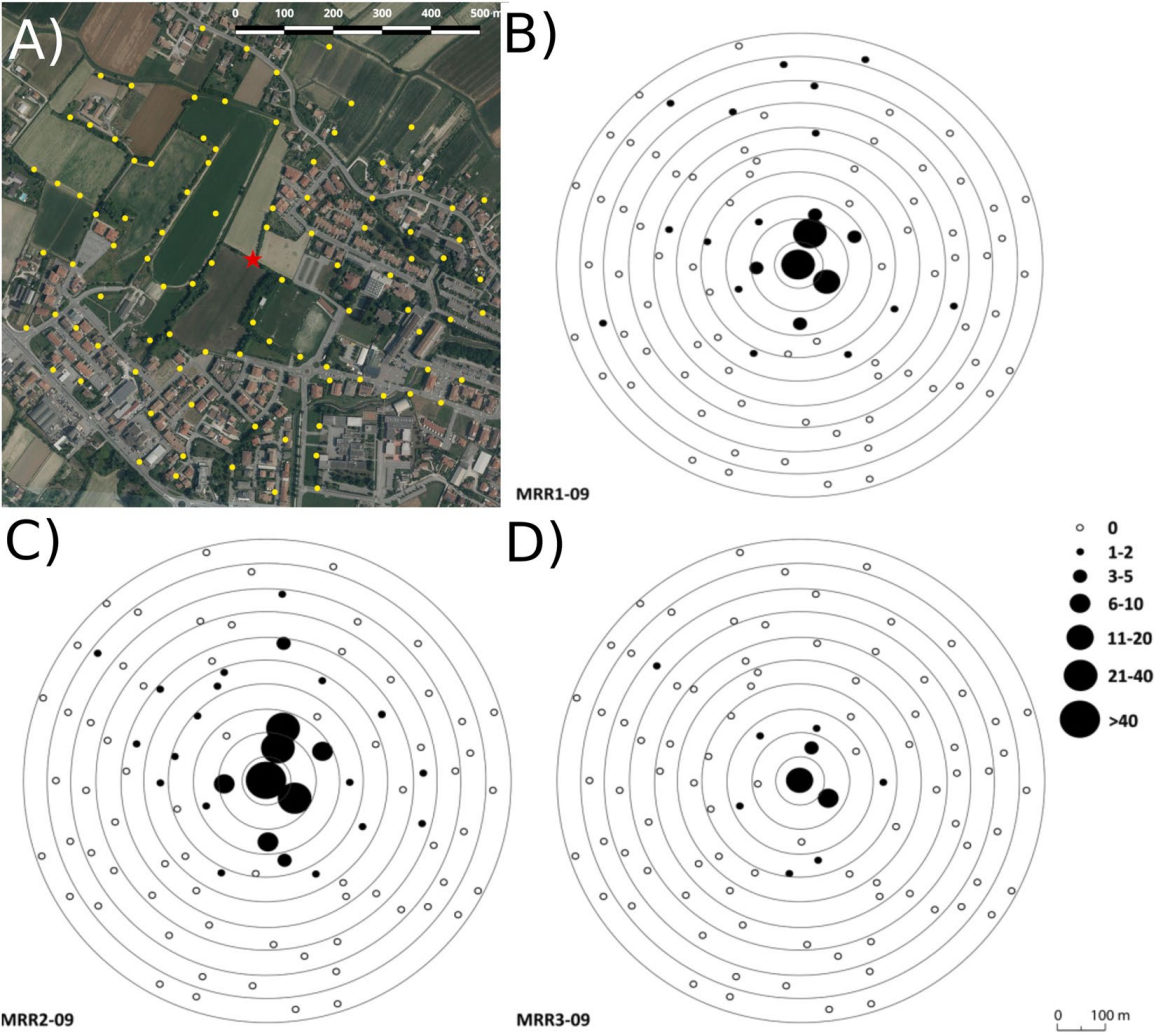
(**A**) Study area in Piove di Sacco (north-east Italy), where 96 sticky traps (yellow dots) were located into concentric annuli of 50 up to maximum of 500 m radius around the release point (red star). Map layout generation were done using QGIS 2.18 (QGIS Development Team (2017). QGIS Geographic Information System. Open Source Geospatial Foundation Project. http://qgis.osgeo.org) free and open source software for geographic information system. The base layer “Regione del Veneto – L.R. n. 28/76 – Formazione della Carta Tecnica Regionale” was obtained from the Veneto Region Geoportal (https://idt2.regione.veneto.it/condizioni_utilizzo_geoportale/) distributed under Italian Open Data License 2.0 (IODL 2.0 http://www.dati.gov.it/iodl/2.0/). (**B–D)** Distribution of recaptured *Aedes albopictus* females in the sticky-traps during the three mark-release-recapture experiments (MRR1, MRR2 and MRR3).

**Figure 2:**
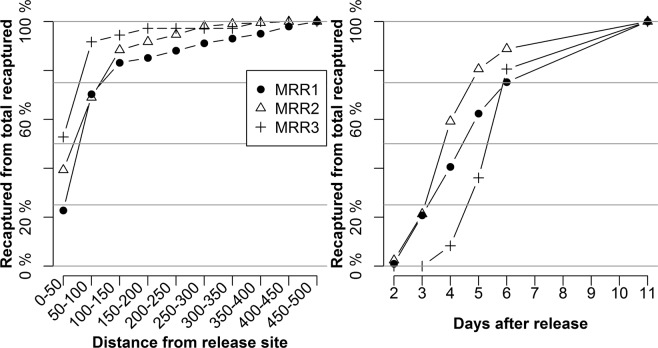
Cumulative proportion of recaptured *Aedes albopictus* females on total of recaptured across the study area (**A**) and during the first 11 days after release (**B**), in the three mark-release-recapture experiments (MRR1, MRR2 and MRR3).

Notably, more than 70% of recaptured marked females were collected within 150 m from release site (Figs 1B–D, 2A; Supplementary Table S2) and during the first 6 days of sampling (Fig. 2B; Supplementary Table S2).

Table 1 summarizes the mean distance travelled (MDT), maximum observed distance travelled (maxODT) and flight range of 90% (FR_90_) and 50% (FR_50_) of marked females in each MRR experiment. Cumulative MDTs were 110 m (days 2–11), 77 m (days 2–16) and 68 m (days 2–16) in MRR1, MRR2 and MRR3, respectively. Daily MDTs were statistically different between the first two MRRs and MRR3 (Mann Whitney test MRR1 vs MRR3: U = 4, P = 0.03; MRR2 vs MRR3: U = 1, P = 0.01), but not between MRR1 and MRR2 (Mann Whitney test U = 27.5, P = 0.19). The maxODTs were 463 m (day-4), 433 (day-5) and 375 (day-6) in MRR1, MRR2 and MRR3, respectively. The flight ranges were always ≤50 m for FR_50_ and ≤252 m for FR_90_ (Table 1) and statistically different among the three MRR experiments (i.e. the interaction effect between log_10_(annulus median distance + 1) and release was significant as shown by a Likelihood ratio test P < 0.01, so the three lines were not parallel between them: MRR1 vs MRR2: t = 4.98, P < 0.01; MRR1 vs MRR3: t = 4.08, P < 0.01; MRR2 vs MRR3: t = −9.06, P < 0.01).

**Table 1:** Mean distance travelled (MDT), maximum observed distance travelled (maxODT) and flight ranges of 90% (FR_90_) and 50% (FR_50_) of recaptured *Aedes albopictus* females in three mark-release-recapture experiments (MRR1, MRR2 and MRR3).

	Days after release	MRR1		MRR2		MRR3	
Daily MDT *(Daily maxODT)*	2	75	*(73)*	95	*(114)*	—	—
	3	80	*(287)*	46	*(275)*	—	—
	4	171	*(463)*	72	*(388)*	58	*(71)*
	5	104	*(436)*	97	*(433)*	35	*(71)*
	6	106	*(333)*	72	*(131)*	78	*(375)*
	7–11	107	*(352)*	121	*(333)*	25	*(168)*
	12–16	—	—	132	*(433)*	25	*(192)*
FR_90_		252		209		177	
FR_50_		50		31		21	

All parameters are expressed in meters.

**Figure 3:**
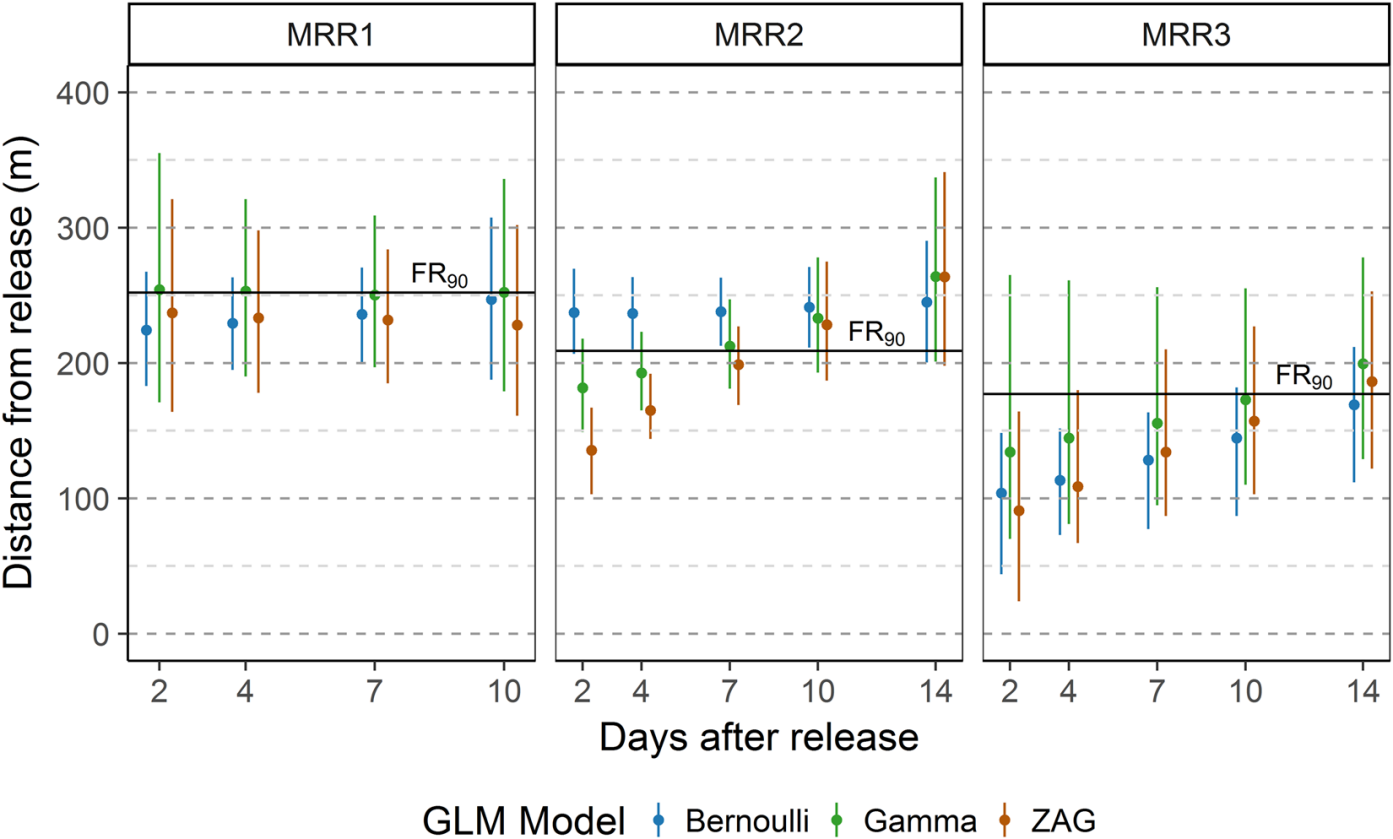
Distance up to which 90% of mosquitoes are expected to travel estimated by three models (Bernoulli GLM, Gamma GLM, Zero Altered Gamma model). On the x-axis the days after release, on the y-axis the distance from the release site. Dots represent the mean distance value; vertical lines represent the 95% confidence intervals obtained by non-parametric bootstrap. The horizontal solid black lines represent the flight ranges of 90% marked *Aedes albopictus* (FR_90_, calculated according to Lillie *et al*.^29^, White & Morris^30^, and Morris *et al*.^31^). Each panel identifies a mark-release-recapture experiment (MRR1, MRR2 and MRR3).

Flight ranges estimates were on average in agreement with results of Gamma GLM and ZAG models, as shown in Fig. 3, which shows model results for the 90% percentiles of the distance up to which mosquitoes are expected to travel, a proportion chose in compliance to allow comparison with the estimated FR_90_. The 90% percentile values reached about 250 m after 2 (MRR1) and 14 (MRR2) days since release (this estimate was not reached during the sampling period in MRR3). On average, the 90% percentile of the distance up to which mosquitoes are expected to fly was stable in MRR1, but it was predicted to increase with time in MRR2 (from <150 m at day-2 to ~250 m at day-14) and in MRR3 (from <100 m at day-2 to ~200 m at day-14), although with shorter estimates and wider confidence intervals due to the lower numbers of mosquito recaptured (Fig. 3).

Result of the Bernoulli GLM model allows to estimate the distance up to which 90% of marked mosquito is expected to be detected. This estimate was stable in MRR1 and MRR2, ranging on average between 170 and 300 m, while a shorter range (between 100 and 200 m) was estimated for MRR3 (Fig. 3). Similar to flight range estimates, also the 50% percentiles of the distance up to which mosquitoes are expected to fly were computed for all three models (see Supplementary Material and Supplementary Fig. S1).

### Effect of fluorescent dye marking on mosquito survival

The survival of marked *Ae. albopictus* females kept under semi-field conditions for the duration of the experiments was shown not to be affected by the fluorescent dyes. In fact, no differences in survival between marked (N = 56, 43 and 43) and unmarked (N = 49, 48 and 48) females were recorded in MRR1, MRR2 and MRR3, respectively (MRR1: log-rank test χ^2^ = 0.15, df = 1, p = 0.70; MRR2: log-rank test χ^2^ = 0.26, df = 1, p = 0.61; MRR3: χ^2^ = 0.10, df = 1, p = 0.76).

### Oviposition pattern under semi-field conditions

**Figure 4:**
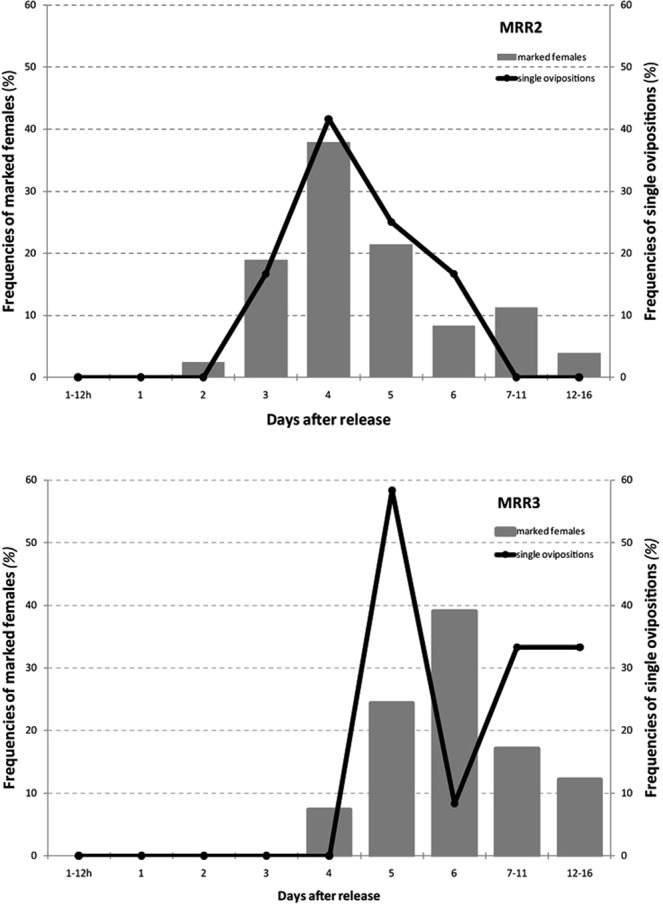
Frequencies of marked *Aedes albopictus* females collected (grey bar) in the second (MRR2, (**A**) and third (MRR3, (**B**) mark-release-recapture experiment *versus* frequencies of ovipositing females kept in semi-natural conditions (single ovipositions, black line) during the same interval.

A positive correlation was observed in MRR2 (Spearman’s rank correlation: r = 0.92; t = 5.38, df = 5, p < 0.01) between the frequency of recaptured *Ae. albopictus* females and the oviposition pattern of blood-fed females simultaneously to the released ones and kept under semi-natural conditions for the entire length of the experiments (Fig. 4). No correlation was observed in MRR3 (Spearman’s rank correlation: r = 0.42; t = 1.05, df = 5, p = 0.34), when a strong thunderstorm and heavy rainfall occurred few hours after the release, likely affecting more the released females than those kept under semi-natural (and “sheltered”) conditions. Consistently with the temperature drop after MRR3 (Supplementary Table S3), the oviposition dynamics of marked females in MRR2 and MRR3 showed a 2-day shift in the peak of oviposition activity.

## Discussion

The first aim of the present work was to study active dispersal of blood-fed *Ae. albopictus* females in a peri-urban habitats in order to confirm or dispute that evidence obtained in a similar study carried out in a highly urbanized study area in Rome^17^ can be generalised and represent a reliable estimate of *Ae. albopictus* female dispersal associated to search of an oviposition site in other temperate regions.

The results obtained showed that 90% of marked *Ae. albopictus* blood-fed females were recaptured within 250 m from the release site (FR_90_ = 177–252 m), a range consistent with estimates from the results obtained in Rome (FR_90_ = 168–236^17^). Moreover, the comparison between the oviposition patterns of females kept under semi-field conditions and the recapture rates in MRR2 supports the hypothesis that the observed dispersal was mostly triggered by the need of *Ae. albopictus* females released at the blood-fed stage to find a suitable oviposition site after blood-meal digestion (Fig. 4). Therefore, 250 m may represent a consistent and possibly generalizable estimate of FR_90_ of blood-fed *Ae. albopictus* females in Italy, and possibly in temperate regions, as this value did not significantly differ in relation to the ecology (peri-urban/rural *vs* urban) or the size (500 m *vs* 250 m radius) of the two study areas tested. However, it is important to highlight that the results obtained are based on mosquitoes reared under optimal conditions, while adult fitness and behaviour in nature are certainly affected by several features such as competition for trophic resources at the larval stage and climatic conditions. For instance, Mori^18^ reported that *Ae. albopictus* emerging from larvae reared under crowded conditions dispersed further than those reared in uncrowded conditions. Also, increased skip oviposition was reported in adults exposed to low-quality/crowded versus high-quality/uncrowded larval habitats^16^. These observations imply that gravid females will move greater distances when larval habitat quality decreases and competition for resources increases. On the other hand, extreme climatic conditions negatively affect survival and dispersal. In fact, compared to results from MRR1 and MRR2 (which were carried out under optimal climatic conditions), results from MRR3 (which was affected by heavy rainfall and a severe drop in temperature) show a much lower recapture rate, a delayed oviposition dynamics between released females and females kept in sheltered cages, and lower-distance dispersal.

The second aim of the study was to model the temporal dynamics of the dispersal, a parameter to our knowledge previously analysed only in a study carried out in Florida showing that the dispersal of gravid *Ae. albopictus* females increased with time, but seemed to stabilize around 90 m from the initial marking site^16^. Our analyses of the temporal patterns of dispersal revealed that the distance travelled by blood-fed females is longer and does not always increase over time. In fact, results from MRR1 showed that the potential exists for a very rapid dispersal, as it is predicted that about 90% of marked mosquitoes have flown within 250 m in 72 hours since release and the remaining 10% have flown even longer (Fig. 3). Both in Padua and in Rome single females were found at the limits of the study areas only 4 days since the release (maxODT Padua = 464 m; maxODT Rome = 290 m). In particular, Bernoulli GLM model showed that the detection probability was not affected by the day of the release in MRR1 and MRR2, suggesting that some individual mosquitoes could rapidly disperse in very few days after release (see Supplementary Table S2, day 4).

The obtained results are very relevant for the planning of control activities to prevent autochthonous transmission of exotic arboviruses in temperate areas, an occurrence which is likely to become recurrent, as testified by the Chikungunya outbreak occurred in central Italy in summer 2017 with almost 500 human infected cases^19^, as well as in France^20^ and Croatia^21^, and by the models predicting future patterns of transmission under climate change scenarios^22,23^. The delayed application of peridomestic/perifocal space spray treatments with insecticides around households where human arbovirus infection has been reported is a critical issue concerning their effectiveness in reducing transmission risk. Despite that, guidelines from the Italian and French Ministries of Health^24,25^ recommend insecticide treatments in a 200 m buffer area around residence of confirmed infected patient independently from the delay between notification and symptom onset. Based on our results and the model’s predictions over time, however, the recommended 200 m buffer does not appear to be sufficient to target 90% of the mosquito population which may have been in contact with the infected case in her/his household. First, the FR_90_ was found to exceed 200 m in some of the MRR experiments carried both in Rome and in Padua. Second, modelling results showed that dispersal can be so rapid that insecticide spraying within a 200 m radius around a potential infected case could fail to reduce below 10% the probability that a potentially infected mosquito escapes the treatment, even if this is carried out after 2–3 days since the arrival of a viremic case (i.e. the shortest plausible time-interval for activation of mosquito control activities after the report of suspect case of arbovirosis to health authorities).

In conclusion, the above results suggest that insecticide treatments aimed to prevent risk of autochthonous outbreaks of exotic arboviruses in temperate regions may fail to reach a non-neglectable part of the potential vector population even if implemented in a 200-m buffer after reports of an infected case to health authorities, considering an average of 6 days delay between symptom onset and detection^26^. Overall, this reinforce the claim that control activities carried out around the place of residence of infected cases are not sufficient to control an emerging arbovirus outbreak, not only due to the importance of outdoor activities in the spread of *Aedes*-borne diseases in temperate countries^27^, but also due to operational constraints in intervening in an adequate area in due time.

## Methods

### Study area

The study area (≈80 hectares) lies within the municipality of Piove di Sacco (Padua province, north-east Italy) and is characterized by either peri-urban or rural habitats (Fig. 1A). The peri-urban habitat is mostly characterized by two-story residential buildings with small gardens and includes football and recreational grounds, small stores, schools, a fuel station and a church. The rural habitat comprises large corn and alfalfa cultivations, small vineyards, horse and sheep pastures, and irrigation canals. A highway delimits the northern and southern extremes of the study area.

### Mark-release-recapture experiments

Three MRR experiments were performed in August 3^rd^ (MRR1) and 24^th^ (MRR2), and in September 9^th^ (MRR3) 2009, with the agreement of Piove di Sacco municipality. Temperature data during the experiments (Supplementary Table S3) were obtained from the ARPAV agrometeorological station of Legnaro (45°20′51N, 11°57′08E), located about 10 km northwest from the centre of the study area (45°18′21″N, 12°01′49″E). Rainfall was absent/very low during all MRRs (≤0.2 mm), except for a few days of moderate rain in MRR1 (i.e. 9.2 mm on day-7 and 3.8 mm on both day-10 and day-11 since release) and a strong thunderstorm (≈170 mm of rain) occurred few hours after release in MRR3 (Supplementary Table S3). Mark-release-recapture experiments were carried out using the same approach described in Marini *et al*.^17^ in Rome.

*Aedes albopictus* females released were obtained from eggs collected by >20 ovitraps located in Padua province. Eggs were left to hatch and larvae were reared in plastic basins (35 × 28 × 8 cm) at larval density ∼1 larva/mL with cat pellets as food (Friskies® Adults). Pupae were transferred to 40 cm cubic cages, where adults emerged and were maintained with 10% sugar solution until the day of release. Eggs, larvae and adults were always kept outdoors in a site sheltered from direct sunlight and wind.

In the morning of each release, 2–4 days-old females were fed with defibrinated fresh bovine blood by a hand-made membrane feeding system^28^. Blood-fed females were transferred into paper-cups closed by a net in groups of 20, and marked by gently dropping orange fluorescent dust (Day-Glo Color Corp., Cleveland, OH, U.S.A.) into the cup. Marked females were then transferred back into the cages and provided with a 5% sugar solution. In the evening, cages were brought by car to the release site in the centre of the study area inside paper boxes. At 10 PM - when *Ae. albopictus* is known to be inactive^2^ - marked females were released by opening the cages, which were left open all night long, in order to leave time to mosquitoes to fly away. Alive and dead individuals found in the cages the following morning were counted and excluded from the total number of released mosquitoes.

Collections of adult mosquitoes were performed using 96 sticky-traps (STs)^29^ located at ground level in sheltered positions and geo-referenced, using a global positioning system device (Garmin GPSMAP 60CSx). In order to have the same collection-power at every distance from the release site, the study area was virtually subdivided into 10 concentric annuli (from 50 to 500 m radius) around the release site, with the number of STs/annulus increased from the centre to extremes of the sampling area to maintain the proportion ST/area to an average density of ≈1 ST/8,200 m^2^ in each annulus (Fig. 1A). All STs were activated by adding ≈500 ml of tap-water and adhesive sheets approximately 12 h after release (day-1); only the ST located on the release site was activated 24 h later (day-2), to avoid collecting individuals which did not disperse immediately after release. Hereafter, the 12 h between release and STs activation are indicated as “day-0”. STs were monitored daily for the first 6 days and at days 11 (MRR1, MRR2 and MRR3) and -16 (MRR2 and MRR3) after release, as described in Marini *et al*.^17^. All collected mosquitoes were counted and morphologically subdivided by genus/species and gender under a dissecting microscope; the presence of fluorescent dust was checked under UV light.

Recapture rates were calculated for each MRR experiment as the proportion of the number of recaptured marked mosquitoes over the total number of released ones. Recapture rates in the three MRRs experiments were compared by a Bernoulli GLM, with the number of mosquitoes recaptured within the first 11 days as response variable and the MRR experiment as covariate. These and the following statistical analyses were carried out using the statistical software R^30^.

To allow direct comparison of the results obtained in the present study and those previously obtained in Rome, dispersal of the released mosquitoes was analysed as in Marini *et al*.^17^, i.e. estimating: (i) the Mean Distance Travelled during the entire sampling period (MDT, a parameter not inherently biased by trap location or size of study area^31–33^); (ii) the daily MDTs for the first 6 days after release; (iii) the maximum Observed Distance Travelled (max ODT), corresponding to the linear distance from the release site to the farthest positive ST (i.e. where at least one marked mosquito female was collected); and iv) the Flight Ranges (FRs) based on the linear regression of the cumulative number of expected recaptures (ERs) from each annulus (x-axis) on the log_10_ (annulus median distance +1). FR_50_ and FR_90_ values were calculated from the equation of regression line as 50% and 90% of the largest ER value, respectively^31–33^. Additionally, the linear regression of the cumulative number of expected recaptures (ERs) was also computed considering the log_10_ (annulus median distance +1), the release and their interaction to test the difference between flight ranges in each release (by comparing the slopes of the three regression lines).

To provide estimates of the distance up to which marked *Ae. albopictus* females are expected to be found conditional to the time of release, dispersal data were further analysed by means of the following three models: (1) a GLM with Bernoulli distribution and logit link to model the probability to detect a marked mosquito conditionally to days and distance from release, and therefore to estimate the probability to detect a marked mosquito at a given distance. The response variable in the model is the presence/absence of marked mosquitoes into the STs, while covariates are the distances of STs from the release site and the days after release; (2) a GLM with Gamma distribution and log link (hereafter Gamma GLM) to model the distance travelled by marked mosquitoes conditional to the days after release and to compute the percentile of the distance travelled by recaptured marked mosquitoes. The response variable in this model is the distance from the release site at which marked mosquitoes were captured; in compliance with the Gamma distribution constrain, only strictly positive distances are considered. Covariate are the days after release; (3) a Zero Altered Gamma model (hereafter ZAG^34^), which accounts for the recaptured mosquitoes into the ST located on the release site (zero distance) and allows to estimate the percentile of the distance at which mosquitoes are expected to be found, weighted for the probability that marked mosquitoes collected actually moved from the release site. Therefore, in this model the distance travelled is modelled conditional to the days after release by means of a two-step model. First, a Bernoulli model with a binary response variable (i.e. value of 0 for mosquitoes recaptured on the release site and of 1 for mosquitoes recaptured in another ST) is applied to evaluate the probability that a marked mosquito moves away from the release site. Second, a Gamma GLM is fitted on distances from the release site at which marked mosquitoes were captured. Covariate was the days after release for both sub-models. In all the three models, confidence intervals for the estimated quantities are computed by non-parametric bootstrap applied to original data.

### Effect of fluorescent dye marking on mosquito survival

Before each MRR experiment, *Ae. albopictus* blood-fed females were removed from the cages by manual aspiration before (“unmarked group”; N = 50) and after (“marked group”; N = 50) the marking procedure described above. Both groups were kept in 25 cm cubic cages kept outdoors in sites sheltered from direct sunlight and wind and provided with water bowl for egg-laying and 5% sugar solution *ad libitum*.

Number of dead mosquitoes was recorded daily for the entire duration of the MRR experiment. Mortality rates in the two groups were analysed using the Kaplan-Meier method and comparing survival curves by Log-rank test.

### Oviposition pattern under semi-field conditions

During MRR2 and MRR3, individual marked blood-fed females (N = 48 and 24, respectively) reared and handled as described above, were kept in plastic cups closed with mosquito net and lined with filter paper, kept in sheltered sites outdoors. Oviposition activity was monitored daily for the entire length of the MRR experiments, by counting the number of eggs deposited on the filter paper in each cup. The correlation between the number of marked mosquito females collected in MRR2 and MRR3 and the number of the ovipositing females during the same intervals was assessed by Pearson’s correlation.

## Supplementary information

Supplementary Information

## Data Availability

All data generated or analysed during this study are included in this published article (and its Supplementary Information Files).
