# Supplementary material for: Estimating Spatio-Temporal Dynamics of Aedes Albopictus Dispersal to Guide Control Interventions in Case of Exotic Arboviruses in Temperate Regions
Source: Sci Rep. 2019 Jul 16;9:10281. doi: 10.1038/s41598-019-46466-4 (PMC6635400; doi:10.1038/s41598-019-46466-4)
Supplement: Supplementary file 1 — Supplementary Information [file 41598_2019_46466_MOESM1_ESM.pdf]

Supplementary Information

ESTIMATING SPATIO-TEMPORAL DYNAMICS OF AEDES ALBOPICTUS DISPERSAL TO GUIDE CONTROL INTERVENTIONS IN CASE OF EXOTIC ARBOVIRUSES IN TEMPERATE REGIONS

Francesca Marini[1,§], Beniamino Caputo[1], Marco Pombi[1], Manuela Travaglio[2], Fabrizio Montarsi[3], Andrea Drago[4], Roberto Rosà[5], Mattia Manica[5], Alessandra della Torre[1*]

[1] Dipartimento di Sanità Pubblica e Malattie Infettive, "Sapienza" Università di Roma, Piazzale Aldo Moro 5, 00185 Rome, Italy
[2] Dipartimento di Biologia, Università di Padova, Viale G. Colombo 3, 35121 Padua, Italy
[3] Istituto Zooprofilattico Sperimentale delle Venezie, Viale dell'Università 10, 35020 Legnaro (PD), Italy
[4] ENTOSTUDIO srl, Viale del Lavoro 66, 35020 Ponte San Nicolò (PD), Italy
[5] Department of Biodiversity and Molecular Ecology, Research and Innovation Centre, Fondazione Edmund Mach, Via E. Mach 1, 38010 San Michele all'Adige, Italy
[6] Center Agriculture Food Environment, University of Trento, 38010 San Michele all'Adige, Trento, Italy

* corresponding author: ale.dellatorre@uniroma1.it; Phone: 0039 06 4969 4268; Fax: 0039 06 4969 4268
§ Current address: Biotechnology and Biological Control Agency (BBCA) Onlus, Via Angelo Signorelli 105, 00123 Rome, Italy

Model results for 50% percentile

The 50% percentile values reached about 100 m after 2, 7 and 10 days since release in MRR1, MRR2 and MRR3, respectively (Figure S1). On average, the 50% percentile of the distance up to which mosquitoes are expected to travel was stable in MRR1, but was predicted to increase with time in MRR2 (from <50 m at day-2 to ~150 m at day-14) and in MRR3 (from <50 m at day-2 to ~100 m at day-14) by the ZAG model, which account for mosquito captured in the release site. The Gamma model instead, highlights that the distance travelled by mosquito leaving the release point is quite high (about 100 m) very early after the release (Supplementary Fig. S1). Result of the Bernoulli GLM model allows to estimate the distance up to which 50% of marked mosquito are expected to be detected. This estimate ranged on average between 0 and 150 m in the three experiments and was affected by the lower number of recaptured mosquito resulting in wide confidence intervals (Supplementary Fig. S1).

Supplementary Figures

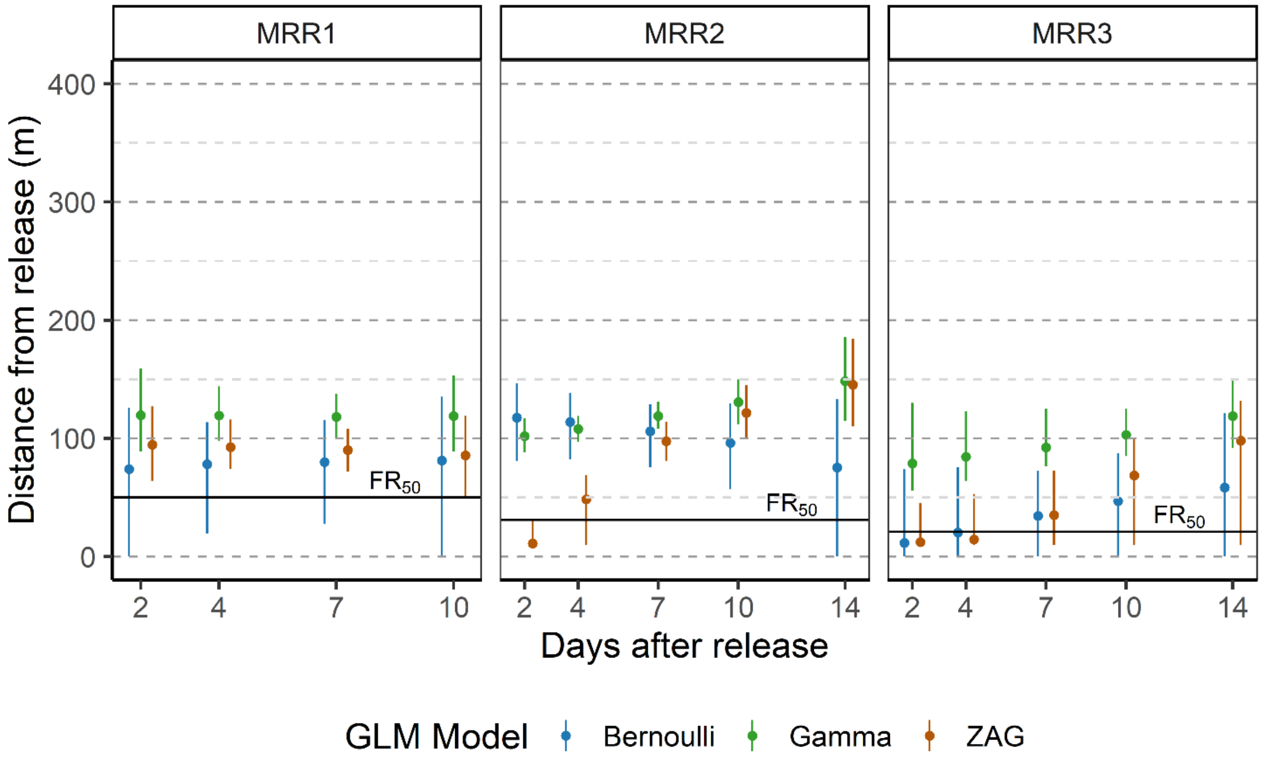

Supplementary Figure S1 - Distance up to which 50% of mosquitoes are expected to travel estimated by three models (Bernoulli GLM, Gamma GLM, Zero Altered Gamma model). On the x-axis the days after release, on the y-axis the distance from the release site. Dots represent the mean distance value; vertical lines represent the 95% confidence intervals obtained by non-parametric bootstrap. The horizontal solid black lines represent the flight ranges of 50% marked *Aedes albopictus* (FR_{50}, calculated according to Lillie et al.[23], White & Morris[30], and Morris et al.[31]). Each panel identifies a mark-release-recapture experiment (MRR1, MRR2 and MRR3).

Supplementary Tables

Parameters	Estimate	SE	Z value	Pr(>|z|)
**MRR1** (intercept)	-2.34	0.10	22.46	<0.0001
**MRR2**	0.43	0.12	3.34	0.0009
**MRR3**	-1.15	0.20	-5.76	<0.0001

Supplementary Table S1 - Binomial GLM estimating the different among the recapture rates after 11 days from release in the three mark-release-recapture experiments (MRR1, MRR2 and MRR3).

Experiment	metres	Days after release†							
		**2**	**3**	**4**	**5**	**6**	**7-11**	**12-16**	***1-16***
**MRR1***	0-50	-	6	3	5	2	7	-	*23*
	50-100	1	9	8	12	8	10	-	*48*
	100-150	0	4	2	3	0	4	-	*13*
	150-200	0	0	1	0	1	0	-	*2*
	200-250	0	0	2	0	1	0	-	*3*
	250-300	0	1	0	0	0	2	-	*3*
	300-350	0	0	0	0	1	1	-	*2*
	350-400	0	0	1	0	0	1	-	*2*
	400-450	0	0	1	2	0	0	-	*3*
	450-500	0	0	2	0	0	0	-	*2*
	0-500	*1*	*20*	*20*	*22*	*13*	*25*	-	*101*
**MRR2***	0-50	-	28	34	13	5	1	1	*82*
	50-100	3	7	25	12	7	7	1	*62*
	100-150	2	1	13	10	5	9	4	*44*
	150-200	0	1	2	3	0	1	0	*7*
	200-250	0	1	1	2	0	2	1	*7*
	250-300	0	1	2	3	0	1	0	*7*
	300-350	0	0	0	0	0	2	0	*2*
	350-400	0	0	1	0	0	0	0	*1*
	400-450	0	0	0	1	0	0	1	*2*
	450-500	0	0	0	0	0	0	0	*0*
	0-500	*5*	*39*	*78*	*44*	*17*	*23*	*8*	*214*
**MRR3***	0-50	-	0	1	8	6	4	0	*19*
	50-100	0	0	2	2	8	2	1	*15*
	100-150	0	0	0	0	1	0	2	*3*
	150-200	0	0	0	0	0	1	2	*3*
	200-250	0	0	0	0	0	0	0	*0*
	250-300	0	0	0	0	0	0	0	*0*
	300-350	0	0	0	0	0	0	0	*0*
	350-400	0	0	0	0	1	0	0	*1*
	400-450	0	0	0	0	0	0	0	*0*
	450-500	0	0	0	0	0	0	0	*0*
	0-500	*0*	*0*	*3*	*10*	*16*	*7*	*5*	*41*

Supplementary Table S2 – Marked *Aedes albopictus* females recaptured during each mark-release-recapture experiment (MRR1, MRR2 and MRR3) in the concentric annuli of 50 up to maximum of 500 m radius around the release site. * - Number of marked *Aedes albopictus* females released: 1,149 (MRR1); 1,600 (MRR2) and 1,210 (MRR2). † - Since between mosquitoes release and STs activation there is an interval of 12h, days indicated have to be reduced by 12 h (e.g. mosquitoes collected on day-2 were actually collected 36h after release instead of 48).

Weather data			Days after release																
			**1**	**2**	**3**	**4**	**5**	**6**	**7**	**8**	**9**	**10**	**11**	**12**	**13**	**14**	**15**	**16**	***tot***
**MRR1**	**T** (°C)	mean	22	24	25	25	26	26	24	24	25	25	24	-	-	-	-	-	***25***
		min	17	16	18	20	22	20	19	19	19	20	20	-	-	-	-	-	***19***
		Max	29	31	31	30	31	32	32	31	32	31	29	-	-	-	-	-	***31***
	**RH** (%)	min	44	33	44	37	35	36	39	49	36	50	49	-	-	-	-	-	***41***
		Max	100	100	91	91	85	90	97	99	100	95	100	-	-	-	-	-	***95***
	**Rainfall** (mm)	tot	0	0	0	0	0	0	9	0	0	4	4	-	-	-	-	-	***2***
**MRR2**	**T** (°C)	mean	24	25	27	27	25	22	21	22	24	25	26	21	19	20	21	21	***23***
		min	18	18	22	22	21	16	15	13	17	21	20	16	14	14	15	17	***17***
		Max	30	30	32	35	31	27	30	32	30	31	33	25	25	26	26	26	***29***
	**RH** (%)	min	35	47	47	35	51	32	26	31	47	41	31	37	25	28	27	30	***36***
		Max	85	93	100	100	97	87	90	100	100	96	89	79	72	77	71	67	***88***
	**Rainfall** (mm)	tot	0	0	0	0	0	0	0	0	0	0	0	0	0	0	0	0	***0***
**MRR3**	**T** (°C)	mean	18	20	21	21	21	21	21	21	20	21	20	20	19	19	20	20	***20***
		min	17	17	16	16	16	18	15	14	16	15	16	15	14	12	14	16	***15***
		Max	19	26	27	28	28	26	27	28	29	27	25	26	27	27	26	26	***26***
	**RH** (%)	min	94	57	49	48	46	49	33	34	29	43	41	39	38	47	51	50	***47***
		Max	100	100	100	100	100	100	96	100	100	100	88	92	98	100	100	100	***98***
	**Rainfall** (mm)	tot	169	0	0	0	0	0	0	0	0	0	0	0	0	0	0	0	***11***

Supplementary Table S3 – Weather data concerning the three mark-release-recapture experiments (MRR1, MRR2 and MRR3), downloaded from the ARPAV agrometeorological station of Legnaro (45°20'51N, 11°57'08E), located about 10 km northwest from the centre of the study area.
